# Acantholytic variant of squamous cell carcinoma of breast: a rare case report

**DOI:** 10.3332/ecancer.2011.214

**Published:** 2011-09-28

**Authors:** HT Kamra, PA Gadgil, SA Chaware, UA Kanade

**Affiliations:** 1 Department of pathology, Government Medical College, Latur-413512, Maharashtra, India

**Keywords:** Pseudoglandular, squamous cell carcinoma, breast, acantholytic

## Abstract

Primary squamous cell carcinoma of the breast is a very rare and aggressive malignancy, and very few have been reported in the literature. Here we report a case of an acantholytic variant of a squamous cell carcinoma of the breast in a 60-year-old woman who presented with a lump in the right breast. Histogenesis is controversial as to whether a pure form of squamous cell carcinoma exists or if these malignancies represent an extreme squamous metaplasia within an adenocarcinoma. Prognosis and management is uncertain due to its rarity and controversy over its definition.

## Introduction

Pure squamous cell carcinoma (SCC), an extremely rare neoplasm, which according to the WHO classification [1] is listed under metaplastic breast carcinoma. Two variants of SCC are acantholytic variant of a squamous cell carcinoma (AVSCC) characterized by lack of cohesiveness of the tumour cells resulting in a pseudovascular or pseudoglandular appearance [2] and adenosquamous carcinoma [3]. The age of patients ranges from 29 to 90 years with a median age of 52 year [4]. The reported incidence is of 0.1% of all ductal carcinomas [5]. Here we report a rare case of an acantholytic variant of squamous cell carcinoma, the rarity of which has been documented by Lee *et al* [6] who noted that up to now only nine cases of AVSCC of breast have been reported.

**Figure 1:**
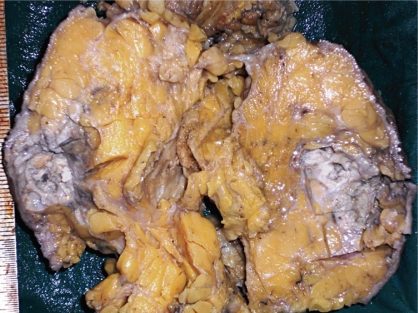
Microphotograph of cut section of the mass showing a cystic area with necrosis in upper portion and a grey white solid area just below it (H&E stain).

**Figure 2:**
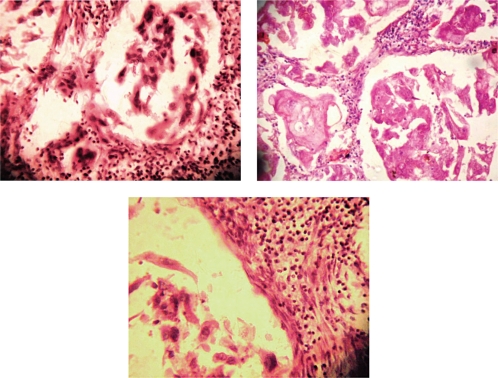
(a) Microphotograph showing malignant squamous cells in pseudoglandular pattern (H&E stain). (b) Microphotograph showing malignant squamous cells in pseudoglandular pattern (H&E stain). (c) Microphotograph showing acantholysis of squamous cells (H&E stain).

**Figure 3:**
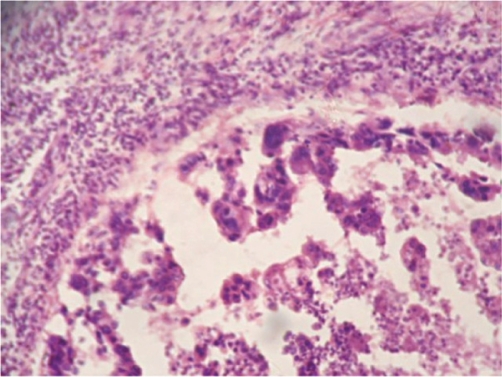
Microphotograph showing intraductal component which showed ducts lined by squamous cells with central region of necrosis (H&E stain).

Case history: A-60-year-old woman presented with 7 × 4 cm lump just below the nipple and areola in her right breast which had developed over the previous two months. There was no retraction or discharge from the nipple and the skin over the lump was normal. Two ipsilateral axillary lymph nodes were enlarged. Left breast examination was normal. Her past and family history were not significant. An ultrasound examination revealed solid hypoechogenic masses with complex cystic components. FNAC yielded 7 ml of dirty fluid from the lump. Air-dried smears were prepared from the centrifuged aspirated fluid and stained with haematoxylin and eosin. The stained smears showed individual malignant squamous cells and loosely cohesive clusters of cells. Cells were polygonal in shape with hyperchromatic enlarged nuclei and coarse chromatin. Keratinous debris was present in the background. A differential diagnosis of primary and metastatic squamous cell carcinoma was made. A total body computed tomography (CT) scan and bone scan, carried out to identify other sites of squamous cell carcinoma in the body, were normal. The patient underwent MRM with ipsilateral axillary clearance. Macroscopically the primary tumour was located 0.5 cm below nipple and areola and measured 7 × 4 × 3 cm. A cross section of the mass showed a cystic area with necrosis in the upper portion and a grey white solid area just below it (Figure 1). Two axillary lymph nodes were isolated. Histopathology showed cells arranged in pseudoglandular pattern, and at places cells were arranged in loose cohesive clusters representing acantholysis (Figure 2a–c). The cells were polygonal, with nuclear pleomorphism, coarse chromatin and dense eosinophilic cytoplasm. Intermingling stroma had lymphocytic infiltration. There was an associated intraductal component which showed ducts lined by squamous cells with central region of necrosis similar to comedo necrosis. The necrotic component was comprised of keratinous debris (Figure 3). Extensive sampling of nipple, areola and skin was carried out but these regions were completely free of neoplastic cells. There was no associated invasive ductal carcinoma or any other feature of metaplastic carcinoma. Only one lymph node was metastatic.

## Discussion

Pure primary squamous carcinoma is a rare and aggressive form of metaplastic carcinoma of breast. Macia and colleagues defined pure squamous cell carcinoma with following criteria [7]

1. No other neoplastic components such as ductal or mesenchymal elements are present in the tumour.
2. The tumour origin is independent of the overlying skin and nipple.
3. Absence of an associated primary squamous cell carcinoma in a second site.

According to Rosen *et al* [8], the presence of *in situ* squamous carcinoma in the ducts is a must for the diagnosis of primary squamous cell carcinoma. They have defined squamous carcinoma as a lesion in which more than 90% of the neoplasm is comprised of squamous carcinoma or its variant. In the case reported here, the tumour had an intraductal component and the carcinoma was comprised of more than 90% of malignant squamous cells. Rosen *et al* [8] have also mentioned that cystic degeneration was associated with primary squamous cell carcinoma and not with metastatic squamous cell carcinoma. This further supported our diagnosis as we aspirated 7 ml of fluid in FNAC, and cystic degeneration was evident macroscopically. The histogenesis of this type of tumour is still unclear. It could represent an extreme form of a squamous metaplasia within an adenocarcinoma or alternatively it may have arisen directly from the epithelium of the mammary ducts [9]. Immunohistochemically, AVSCC is characterized by high proliferative activity, an uncommon cytokeratin expression profile, reduced E-cadherin staining and overexpression of p53 and the epithelial growth factor receptor (EGFR) [10]. In our case, immunohistochemisry was positive for high molecular weight keratin.

Arrangoiz *et al* [4] have reported that axillary lymph node metastases occur rarely and when they do are usually associated with metaplastic squamous cell carcinoma arising as an invasive ductal carcinoma. In this case study, one of the two lymph nodes also showed metastasis with morphological features similar to tumour in breast.

Prognosis appears to be dependent on tumour size and tumour stage but well-documented reports are still lacking. It is hard to tell whether the amount of the acantholytic component affects the prognosis or not, because previous reports did not describe the exact proportion of each component in acantholytic variant of squamous cell carcinoma [2].

## Conclusion

Acantholytic variant of squamous cell carcinoma, a newly defined entity is very rare and aggressive in nature. Such tumours should be distinguished from metaplastic adenocarcinoma with squamoid differentiation since there is no effective adjuvant or neoadjuvant therapy available for its treatment. In the case presented here, the patient was referred to another centre for treatment as she was also triple negative for receptor expression and so we could not follow-up the patient.
